# Yogurt, in the context of a healthy diet, for the prevention and management of diabetes and obesity: a perspective from Argentina

**DOI:** 10.3389/fnut.2024.1373551

**Published:** 2024-04-15

**Authors:** Sergio Britos, Andrea F. González, Florencia Flax Marcó, Mónica Katz, Jacqueline Schuldberg, María Elena Torresani, Gabriel Vinderola

**Affiliations:** ^1^Medical Sciences Faculty, Pontifical Catholic University of Argentina, Buenos Aires, Argentina; ^2^Department of Alimentation, Dr. C. Bonorino Udaondo Gastroenterology Hospital, Buenos Aires, Argentina; ^3^Ministry of Health, Government of Buenos Aires Autonomous City, Buenos Aires, Argentina; ^4^Faculty of Medical Sciences, Favaloro University, Buenos Aires, Argentina; ^5^Villa Urquiza Women and Children Medical Center, Buenos Aires, Argentina; ^6^Instituto de Lactología Industrial (CONICET-UNL), Faculty of Chemical Engineering, National University of Litoral, Santa Fe, Argentina

**Keywords:** yogurt, prevention, management, obesity, diabetes, Argentina

## Abstract

Diabetes is a non-communicable chronic, but preventable, disease whose occurrence is related to unhealthy lifestyles, including inadequate diet. Obesity is a risk factor for diabetes. In Argentina, 12.7% of the population is living with diabetes. In this work, we aimed at giving a perspective on the role of yogurt, as part of a healthy lifestyle, for the prevention and management of obesity and diabetes. The intake of yogurt declined in the last decade in Argentina. In the context of the global diet, the contribution of a moderate increase of yogurt consumption has the potential to improve up to 10% the nutritional density of the Argentine population’s diet, given its present low diversity and wide gaps in nutritive foods. The consumption of yogurt can be beneficial in the prevention and management of obesity and T2DM. The ready availability of yogurt and its easy introduction to diverse diets suggests that educating the general public to incorporate this fermented milk as part of a healthy diet may potentially contribute to improved public health through prevention of NCDs and the costs associated with them.

## 1 Introduction

Non-Communicable Chronic Diseases (NCCDs) are long-term preventable diseases with slow progression and high prevalence worldwide. NCCDs include diabetes, cancer, chronic respiratory diseases, and cardiovascular diseases, among others. Most risk factors for these diseases are related to unhealthy lifestyles like inadequate diet, sedentarism, tobacco and excessive alcohol consumption (1). Around 1 billion people are overweight worldwide, and every year 2.6 million people die from this disease. It is estimated that more than 80% of ischemic heart diseases, strokes, and diabetes, as well as more than a third of cancers, could be prevented through changes in people’s behavior regarding risk factors (2, 3). In high-income countries, 9 of the 10 main causes of death were related to this group of diseases, while in low- and middle-income countries, almost 8 out of 10 deaths occurred from them^1^. In the Americas, NCCDs are the cause of three out of four deaths, and 34% of them are premature, they occur in people between 30 and 60 years old. NCCDs have a considerable economic impact, not only due to the cost of medical care but also due to the loss of people’s productivity since a quarter of deaths from these causes occur in people under the age of 60. Policy measures should focus on improving the efficiency of health care through efficient interventions with an appropriate selection of priorities. Providing timely, high-quality diagnostic and treatment in primary care services has been shown to prevent acute deterioration, progression or complications in people living with these conditions. Likewise, it is necessary to understand that NCCDs involve multifactorial approaches that in many cases exceed the response capacity of the health sector alone (4).

In Argentina, and since 2004, the National Survey of Risk Factors (NSRF) allowed us to know the temporal trends and prevalence of the main risk factors. Some results of the fourth NSRF (5) shows that 66.1% of the population was overweight (52% of them obese) and 12.7% had diabetes or altered blood glucose. Both indicators increased when compared to previous surveys. The prevalence of moderate-high intensity physical activity was 44.2% lower than in the previous survey. Consumption of fruits and vegetables remained very low: only 6% of the population consumes the recommended amount of five portions of fruits and vegetables a day, with no changes when compared to previous reports, and 22.2% of the population consumed tobacco, a habit that has been steadily decreasing since the first NSRF in 2009.

Yogurt may be useful for weight management programs. Its consumption is associated with beneficial changes in anthropometric biomarkers. Several cross-sectional studies showed that yogurt consumers have a significantly lower BMI compared with non-consumers. Women who consumed at least one serving of yogurt a day had significantly lower BMI compared with those not consuming yogurt (6). There is also accumulating evidence suggesting that yogurt consumption is related to several health advantages, including the prevention of osteoporosis, diabetes, and cardiovascular diseases, as well as the promotion of gut health and immune system modulation (7). The aim of this article was to give a perspective on the role of yogurt intake, as part of a healthy lifestyle, for the prevention and management of diabetes and obesity.

## 2 Healthy eating pattern and diet quality in Argentina

Maintenance of a healthy eating pattern is key in the prevention of NCCDs. A healthy diet is based on dietary patterns that promote health and prevent diseases, providing adequacy, without excess, of nutrients and substances from nutritious foods, avoiding the consumption of substances harmful to health. Foods are nutritious when they provide beneficial nutrients and minimize the presence of elements such as sodium, saturated fats, sugars and antinutrients (8). The preponderance of nutritious foods, the proportionality and (broad) diversity of different food groups determine quality levels of the total diet.

In recent years, different studies provided evidence about the relationship between diet and disease prevention. Different eating patterns have been searched for their relationship with health. The best known are the Mediterranean Diet Pattern, the DASH Diet (Dietary Approach to Stop Hypertension) or the Planetary Health Diet (9). All of them recommend a diverse diet, with plenty of fresh vegetables and fruits, legumes, whole grains, nuts, moderate amounts of dairy products, fish and eggs, vegetable oils (including olive) and low amounts of red meat. Such diet exhibits high levels of nutritional quality when measured with different criteria such as the Healthy Eating Index (HEI) originated in the United States Department of Agriculture or the Nutrient Rich Food Index, from the University of Washington (10).

Among dairy products, yogurt is one of the most consumed fermented foods (11). In addition to providing proteins and numerous nutrients in a balanced proportion with its low caloric density, yogurt provides the benefits of its viable bacteria (*Streptococcus thermophilus* and *Lactobacillus delbrueckii* subsp. *bulgaricus*). The role of fermented foods such as yogurt is increasingly considered in dietary guidelines, both for its contribution to nutritional quality: each 100 g of yogurt in a healthy diet of 2000 kcal daily is responsible for 5% of the overall quality and twice as much in the case of unhealthy diets, as for the complementary role of fermentation and the contribution, when present, of probiotics (commonly specific strains of *Lacticaseibacillus casei*, *Lacticaseibacillus paracasei* subsp. *paracasei* or bifidobacteria).

In Argentina, improving nutritional quality is a great challenge for public food policies. According to recent studies (12), the proportion of households whose dietary pattern has a high nutritional density does not exceed 11% and food gaps (difference between apparent consumption and healthy recommendations) are on average 60% in high nutrient density vegetable foods and 48% in dairy products. Progressive reduction of these gaps, also reducing excesses in starchy foods and meats and occasional products, would improve the present low-nutritional density of the Argentinian diet, and would increase its diversity.

## 3 Yogurt as a nutrient-dense food matrix

Foods are commonly associated with nutrients such as proteins, fats and carbohydrates, and other components that appear in nutrition labels. Less known is that, in a product, nutrients are neither homogeneously dispersed, nor in a free form in many cases, but as part of complex microstructures. Evidence has given a great importance to the structure of foods and its relationship with physical, sensorial, and nutritional properties (13). Foods behave according to their own matrices and the type of food matrix directly affects the dynamics of digestion and absorption of food compounds in the gastrointestinal (GI) tract. Diet is the main factor affecting the composition and function of the GI microbiota (14).

Dairy products properties, including health benefits, differ from those of the milk of origin, if fermented. Consumption of yogurt and other fermented products is associated with improved health (15). The contribution of yogurt to the intake of nutrients such as calcium and proteins is substantial, and favorably influences the total daily intake of them. Yogurt consumers are characterized as healthier in their dietary habits and comply with the recommendations of dietary guidelines (16, 17). Yogurt consumption is associated with a healthy eating pattern and lifestyle (18). In this sense, yogurt consumers ingest more essential nutrients, such as minerals (calcium, potassium, magnesium, zinc), vitamins (B2, B12, D) and proteins, and less fat. Regarding food intake, children who frequently consume yogurt have an overall healthier diet. Specifically, they consume more fruits, whole grains, and milk, which indicates a better nutritional profile. Regarding eating habits, yogurt consumers usually eat less fried foods, processed and red meats, pizzas, soft drinks, or alcohol. In addition, yogurt is a versatile matrix that allows adding the beneficial effect of other foods such as whole cereals and fruits. Their combination may provide probiotics, prebiotic fibers, high-quality proteins, important fatty acids and a mixture of vitamins and minerals that have the potential to exert synergistic effects on health. This makes yogurt a recommendable snack considering its association with healthy dietary patterns (19).

## 4 Yogurt intake in Argentina: dietary guidelines and nutrition-economics

According to the consulting agency Kantar Worldpanel, in Argentina, yogurt consumption is presently around 4 kg/person/year. It was around 10 kg/person/year in 2012 according to data presented in Dietary Guidelines for the Argentinian Population^2^. In our country, 9 out of 10 households buy yogurt at least once a year and 53% of households buy both skim and whole yogurts. However, in households where only one variety is purchased, the full-fat version predominates. Consumers buy yogurt about 11 times a year, and in each purchase, they get around 1.16 liters. France is the main consumer of yogurt (45 liters per capita per year). In Argentina, yogurt consumption is reducing, as the category has declined 44% in the last 10 years, driven by both full-fat and skim versions^3^.

In the Dietary Guidelines for the Argentinian Population, yogurt is not indicated as a source of live beneficial bacteria. Hill et al. (20) found that an additional 100-g intake of microbe-containing foods, as yogurt, was associated with a lower systolic blood pressure, C-reactive protein, plasma glucose, plasma insulin, triglyceride, waist circumference and BMI, and a higher level of high-density lipoprotein cholesterols.

In dietary guidelines from north American and European countries, yogurt is recognized for its several health benefits, related to weight management, reduced hyperglycemia, reduced hypertension, protective properties in the gut and the contribution to maintain a healthier microbiota (21–23).

An economic model for the use of yogurt in T2DM risk reduction was developed in the UK. The model predicted that increasing average yogurt consumption by adults by 100g daily could result in 388,000 fewer people developing T2D in the next 25 years. This could save the country’s health system £2.3bn in direct T2DM treatment costs (24). In the US, it was demonstrated that increasing yogurt consumption among adults has the potential to provide billions of dollars in savings (25).

## 5 Yogurt for the prevention and management of diabetes

According to the fourth survey of risk factors in Argentina (2019) in adults, 12.7% of people had diabetes or altered blood glucose (5). Type 1 diabetes mellitus (T1DM) remains the most prevalent form in children, whereas Type 2 diabetes mellitus (T2DM) is estimated to occur in 1 in 3 of new diagnoses of diabetes in children worldwide. There is no data about their incidence in Argentina, neither in children nor adults. Consumption of low-fat yogurt has been associated with a lower risk of developing T2DM (26). This is thought to be due to the simultaneous action of milk proteins, calcium, magnesium, vitamin D and the low glycemic load of yogurts. In addition, certain fatty acids may also be beneficial for the control of T2DM. While even-chain SFA such as myristic, palmitic, and stearic were positively associated with the incidence of T2D, odd-chain SFA such as pentadecanoic and heptadecanoic were inversely associated with the incidence of T2D. This effect according to authors could be due to *de novo* lipogenesis that induces the synthesis of even-chain SFA (27).

However, the association between dairy products consumption and T2DM depends on the type of dairy product and its fat composition, as well as the initial glycemic status of consumers (27). A recent meta-analysis of 14 studies conducted in the United States, United Kingdom, Netherlands, Spain, Australia, and Japan that included 483,090 people found a 7% reduction in the risk of T2DM for each increase of 50 g of yogurt consumed daily (28). The PREDIMED study evaluated the association between the consumption of dairy products and the risk of T2DM in 3,454 non-diabetic Spanish older adults. Dairy product consumption was assessed at baseline and annually with food frequency questionnaires for 4.1 years. After multivariate adjustment, total dairy product consumption was inversely associated with risk of T2DM (OR: 0.68, 95% CI: 0.47–0.98, *p* = 0.040). This association seemed to be attributed to low-fat dairy.

The effect of milk proteins on the release of intestinal hormones such as gastric inhibitory polypeptide (GIP) and glucagon-like peptide (GLP-1) was postulated as a possible mechanism of action (29–31). Both peptides induce insulin secretion and slow nutrient absorption. However, this hypothesis is weakened by the similarity of milk proteins in both low-fat and high-fat milks. Yogurt could due its beneficial effect to the modulation of glucose metabolism (32), and, in addition, to its low glycemic index (GI) (33, 34). Thus, the different authors conclude that the consumption of protein-rich dairy products may be a useful strategy to reduce the risk of developing T2DM and other metabolic disorders, including obesity.

The glycemic and insulinemic impact of plain and sweetened yogurts were compared (14), observing that the 43 plain yogurts assessed had a lower GI than the 50 sweetened ones. It seems that this difference is not explained by sugar *per se*, but rather by the higher protein/carbohydrate ratio in natural yogurts. Different mechanisms were proposed to explain the relationship between the reduced risk of suffering from DM2 and the consumption of fermented dairy products (35). First, because the fermentation process decreases the bioavailability of carbohydrates by hydrolyzing them and forming organic acids and polysaccharides. Second, the presence of menaquinones (vitamin K2) synthesized by animal tissue, although it can also be of microbial origin. Third, the probiotic bacteria present in some yogurts showed to improve the lipid profile, cholesterol concentrations and antioxidant status in individuals with T2DM. Finally, the presence of certain organic acids in foods, whether produced by fermentation such as lactic acid in yogurt, or added, can reduce postprandial glycemia and insulinemia (36). For example, myristic acid was associated with improved glucose homeostasis (37), and plasma levels of very long-chain fatty acids were inversely correlated with disease incidence (27).

## 6 Yogurt for the prevention and management of obesity

In Argentina, 6 out of 10 adults live with excess weight or obesity and more than 40 percent of children and adolescents are overweight or obese. Childhood obesity is an open window to the emergence of chronic diseases such as cardiovascular diseases and T2DM^4^. Globally, more than two billion adults live with excess weight and more than 850 million suffer from obesity^5^.

Obesity is an adiposity-based chronic disease. The excess of fat increases the risk of insulin resistance, T2DM, metabolic liver disease, several types of cancer, obstructive sleep apnea, and cardiovascular disease (38). Body weight is regulated by several hormonal, neuronal and metabolic mechanisms. The intestinal microbiota (IM) exerts direct effects on the consumption, digestion, and metabolism of food (39). Gut dysbiosis is considered a reduction in abundance and diversity of the gut microbiota members, with the reduction of beneficial species and proliferation of potential pathobionts (40). It has been pointed out that an indicator of dysbiosis in obesity is the decrease in Bacteroidota/Bacillota ratio (formerly Bacteroidetes/Firmicutes) (41). However, a meta-analysis disregarded that statement (42). Gut microbiota can modulate metabolism through abundancy and diversity of certain bacteria that may facilitate energy storage and may alter metabolic pathways leading to obesity (43, 44). There are several mechanisms proposed to explain the association between dysbiosis and obesity. One is the so-called “colonic rescue”, where the microbiota in obese people harvests energy more effectively and may manipulate host gene function leading to increased adiposity, aggravation of inflammatory mechanisms, metabolic endotoxemia, and metabolic dysfunction (45). Another mechanism is the regulation of energy balance. On the one hand, IM affects the production of incretins which increase satiety, neurotransmitters that regulate emotions and metabolites such as bile acids involved in the modulation of caloric expenditure (46, 47). A third mechanism is the capacity of the microbiota to modulate fat storage. The IM and its metabolites regulate LPL-mediated lipolysis, leading to changes in triglycerides (TG). Additionally, IM inhibits LPL through the “fasting-induced adipose factor” (FIAF), which reduces the ability of adipocytes to absorb TG (48). A deeper insight into the mechanisms underlying the connection between the IM and the microbiome has been recently published (49). In addition, a recent work reviewed the underlying mechanisms that connect fermented foods, like yogurt, and gastrointestinal health (50). However, there is a gap in the knowledge of the mechanisms that connect yogurt, the microbiome and obesity.

Epidemiological and clinical evidence (51, 52) suggest that yogurt is involved in the control of body weight and energy homeostasis and may play a role in reducing the risk for obesity partly via the replacement of less healthy foods and its diverse food matrix components, and, in some cases, probiotics, with effect on appetite control, energy balance and different anthropometric biomarkers such as body mass index (BMI), waist circumference (WC) and body fat (BF) (53).

Epidemiological studies suggest that yogurt may be useful for weight management. Several cross-sectional studies showed that yogurt consumers have a significantly lower BMI compared with non-consumers. Women who consumed at least one serving of yogurt had a significantly lower BMI compared with those consuming no yogurt (6, 54).

One meta-analysis indicates that yogurt consumption would reduce the overall obesity and abdominal obesity. In addition, long term consumption of yogurt may contribute to some obesity-related anthropometry change (55). Using data from the NHANES 2007–08 for women, among yogurt eaters, BMI, WC, and BF were lower than in non-yogurt eaters (56).

In a retrospective cohort study, high yogurt consumers gained significantly less weight compared with low yogurt consumers, and a significant association between yogurt consumption and decreasing weight gain was found (18). In relation to BF and specifically abdominal fat, several cross-sectional studies reported that yogurt consumers had significantly less body fat compared with non-yogurt consumers, with significant inverse associations between yogurt consumption and total body and abdominal fat (57).

Another important effect is the impact of high yogurt consumption on the risk of developing weight gain and obesity. In fact, yogurt consumption is associated with a lower risk of overweight/obesity. Perhaps, yogurt consumption beyond its matrix components, is linked to healthy eating and lifestyle, which are, in turn, likely to be linked to positive weight-related outcomes (58, 59).

In an increasingly obese world, it would be of value for public health strategies to use a simple, inexpensive, and commonly consumed food that can assist to improve weight-related outcomes. The availability of yogurt, its safety profile and its easy introduction to most eating patterns suggest that educating the public to consume yogurt as part of a balanced and healthy diet may potentially contribute to improved public health. Nevertheless, more well-designed large community-based studies are needed to provide proof of principle of the isolated effect of modulating specific microbiota components on the prevention of weight gain and treatment of the obesity pandemic.

## 7 Conclusion

The intake of yogurt declined in the last decade in Argentina. In the context of the global diet, the contribution of a moderate increase of yogurt consumption has the potential to improve up to 10% the nutritional density of the Argentine population’s diet, given its present low diversity and wide gaps in nutritive foods. The consumption of yogurt can be beneficial in the prevention and management of obesity and T2DM. The ready availability of yogurt and its easy introduction to diverse diets suggests that educating the public to incorporate yogurt as part of a healthy diet may potentially contribute to improve public health through prevention of NCDs and the associated costs.

## Data availability statement

The original contributions presented in this study are included in this article/supplementary material, further inquiries can be directed to the corresponding author.

## Author contributions

SB: Writing – original draft, Writing – review and editing. AG: Writing – original draft, Writing – review and editing. FF: Writing – original draft, Writing – review and editing. MK: Writing – original draft, Writing – review and editing. JS: Writing – original draft, Writing – review and editing. MT: Writing – original draft, Writing – review and editing. GV: Writing – original draft, Writing – review and editing.

## Footnotes

1
https://www.who.int/es/news-room/fact-sheets/detail/the-top-10-causes-of-death

2
https://bancos.salud.gob.ar/recurso/guias-alimentarias-para-la-poblacion-argentina-manual-de-aplicacion

3
https://agroempresario.com/publicacion/1000/el-yogur-creciendo-en-el-consumo/

4
https://www.indec.gob.ar/indec/web/Nivel4-Tema-4-32-68

5
https://www.who.int/news-room/fact-sheets/detail/obesity-and-overweight
